# Design and Implementation of a Walking Stick Aid for Visually Challenged People

**DOI:** 10.3390/s19010130

**Published:** 2019-01-02

**Authors:** Nilima Sahoo, Hung-Wei Lin, Yeong-Hwa Chang

**Affiliations:** 1 Department of Electrical Engineering, Chang Gung University, Taoyuan City 333, Taiwan; nilimasahoo263@gmail.com; 2 Department of Electrical Engineering, Lee-Ming Institute of Technology, New Taipei City 243, Taiwan; kinglin@yahoo.com

**Keywords:** Raspberry Pi, PIC, ultrasonic sensor, GPS, emergency APP, database

## Abstract

Visually challenged people (VCPs) face many difficulties in their routine life. Usually, in many cases, they need to depend upon others, which makes them unconfident in an unfamiliar environment. Thus, in this paper, we present an aid that helps in detecting obstacles and water puddles in their way. This system comprises a walking stick and Android-based applications (APPs). The walking stick is embedded with Raspberry Pi and programmable interface controller (PIC) as a control kernel, sensors, a global position system (GPS) module, and alert-providing components. Sensors help to detect obstacles, and the VCP is informed through vibrations or a buzzer according to the obstacle detected. The GPS module receives the coordinates of the VCP’s location, and the location can be tracked by parents using an APP. Another important APP is used, called an emergency APP, by which the VCP can communicate with parents or friends immediately by just shaking his/her cell phone or pushing the power button four times in 5 s in panic situations. We used fewer components to make the device simple, lighter, and cozy with very good features. This device will help VCPs to live an independent life up to some extent (with security), which ultimately will increase their confidence level in an unknown environment.

## 1. Introduction

Eyes, organs of vision, are an important part of human physiology, as they have the ability to receive and process visual detail to the brain. In addition, they play a vital role in the lives of humans, because 83% of information from the environment is obtained through the eyes [1]. However, there are several people in the world who are deprived of this. There are a number of severe disabilities, of which blindness is one of them, in which a person needs to face many problems despite a number of technological advancements. Blindness is a state of condition where the individual is unable to see and has no light perception. Blindness also refers to those who have so little vision that they use other senses as vision substitution skills. Thus, the visually challenged considers the person who has total vision loss or partial vision loss.

According to an 11 October 2017, report from the World Health Organization (WHO) [2], an estimated 253 million people are living with vision impairment, out of which 36 million are totally blind and 217 million have moderate to severe vision impairment. This means chronic eye diseases are the leading cause of vision loss, which approximates 85%. Out of the total percentage of vision-impaired, 81% of people are aged 50 years or above, and the remaining 19% are people under the age of 15 years. Most blind people belong to low income or developing countries, with most in Africa and Asia. From recent statistics, it is expected that by the year 2050, the vision impairment percentage could triple due to rapid increases in population and aging. By considering these factors (age and financial condition), we tried to streamline the problem defined above, and we designed a structure that is lighter in weight, cost effective, and not complex in structure, so that it is easy to carry and affordable to most visually challenged people.

In this modern technology era, the explosion of new and innovative technology provides many opportunities for everyone to live a more comfortable life. For blind people, many aids have been developed by many researchers to provide an independent life. Some of them have many new features but are expensive, so most visually challenged people (VCP) cannot afford these types of devices. On the other hand, the less expensive aids have shortcomings of certain necessary features. Our proposed device may not be attractive-looking, but our focus was to reach financially weaker VCPs so they can use it and benefit from it. This proposed device has certain features that are helpful for VCPs in moving around their surroundings independently. The walking stick is embedded with two types of sensors, one to detect obstacles at the ground level, as well as for upper body parts from a certain distance. It is preset according to the VCP’s convenience, so that the user can avoid obstacles in time without collision. Another sensor detects small water puddles or wet surfaces in the way of the VCP, which is helpful to the user in avoiding sliding. In addition, the system is incorporated with a GPS module that receives the latitude and longitude of the VCP’s location and sends it to a database for future purposes. Thus, an application (APP) was created that helps the parents of the VCP to track their child in real time. Another APP is used by the VCP that helps to contact parents or guardians easily and immediately in any emergency situation. To recapitulate, VCPs, as well as their parents, feel safe and relaxed, as they can contact each other without any difficulties.

## 2. Related Works

A large number of research works are being performed by various researchers to provide an efficient navigation aid for blind persons. Initially, they just depended upon others for their basic needs and mobility. After that, many blind persons used the traditional white cane and trained dog as assistance to guide their path [3,4], but they have certain limitations. Although the white cane is inexpensive, it cannot detect obstacles accurately, and it can only detect obstacles by touch, so the user may get less time to react to situations, which is very dangerous for the user. A trained dog is expensive, and though it is an animal, it may have the chance of getting hurt or sick. Thus, these solutions are not so efficient. The following subsections describe and discuss some of previous work based on features and technology either of only obstacle-detecting electronic travel aids (ETAs) or obstacle detectors combined with position locators and communication techniques.

### 2.1. Obstacle-Detecting ETAs

Obstacle-detecting ETAs are designed to detect obstacles in the way and to alert a VCP about detecting obstacles to avoid a collision.

Sharma et al. [5] proposed a system called “virtual eye” that helps to improve the mobility of a VCP in a particular area. The system consists of a wearable hat, a mini-hand stick, and shoes, and the components used in this system are ultrasonic sensors for obstacle avoidance, an Arduino microcontroller for processing the signal received by the sensors, and Raspberry Pi as a speech synthesizer to inform the VCP about detected obstacles. The proposed system can detect obstacles in the front, backside, left, and right directions of the user.

Mocanu et al. [6] proposed a wearable device for efficient obstacle detection as well as recognition using sensors, computer vision, and machine learning techniques. The system uses four ultrasonic sensors, Bluetooth, an Arduino microcontroller, and a smartphone. The sensors and smartphone camera collect information about the obstacles and send it to the obstacle recognition module to understand the presence of obstacles in the surrounding scene. A K-mean clustering algorithm was applied for identifying dynamic objects, and a support vector machine (SVM) classifier with *CHI_Square* kernel was used for training and prediction. Accordingly, it generates a feedback signal for the VCP. The proposed system provides a better solution for obstacle detection, as it integrates both sensors and machine learning techniques, but it can only detect obstacles up to the waist level.

Yi et al. [7] designed a blind guide crutch based on ultrasonic distance measurement for obstacle detection. It consists of three ultrasonic sensors for detecting and avoiding obstacles overhead, in front, in the right-front, and in the left-front. A STC15F2K60S2 microcontroller is used to control the whole system and processes the signal between the ultrasonic transmitting and receiving module to obtain distance information. Voice and vibration modules are used as feedback modules. The system has the capability of detecting obstacles in multiple directions.

Mohammed A. Therib [8] proposed a system that comprises a blind stick in order to help blind people avoid collisions with obstacles. It consists of an Arduino microcontroller and two SRF06 ultrasonic sensors: One sensor is fixed at an angle of 40 degrees on the stick to detect stairs or holes, and another sensor is used to detect obstacles such as a wall or any persons in front of the blind person. A moisture sensor is used to detect wet surfaces or ponds, and a vibration motor and buzzer are used as an alarm. The system can detect obstacles, holes, stairs, and moist surfaces.

Saaid et al. [9] developed a prototype of a stick, called a Radio Frequency Identification Walking Stick (RFIWS). It helps a blind person to walk on the sidewalk and avoid falling off of the sidewalk by calculating the distance between the blind person and the sidewalk border. A radio frequency identification (RFID) tag, RFID reader, middleware, and a database are the major components of RFID technology. In this system, the RFID tags are placed at the center of the sidewalk at a certain distance from each other. Thus, when the VCP gets closer to the border, the reader attached to the walking stick detects the frequency from the tag, and vibrations are produced to alert the user. The system is reliable, as RFID technology provides an accurate level of detection, but for this, a lot of RFID tags need to be placed in many places, and each tag has a specific working range, and hence this requires a lot of testing. In addition, if any tag is covered by anything, then it may not work properly [10].

### 2.2. Hybrid ETAs

These ETAs are designed with obstacle detection sensors along with either localization or communication technology. Thus, the systems provide information about the obstacles encountered and the VCP’s location, and also the VCP can contact parents or guardians.

Dhod et al. [11] proposed a prototype of a global position system (GPS) and global system for mobile communication (GSM)-based navigational aid called a “smart white cane” that helps visually impaired people in easy navigation. The system consists of IR sensors for obstacle detection, a water detection sensor, a playback recorder integrated circuit (IC) to provide instructions about the presence of obstacles, a GPS module, and a GSM for finding location and sending SOS messages to family members, respectively. 

Bharambe et al. [12] proposed a prototype named “substitute eyes for the blind”, in which the embedded system mainly helps in obstacle detection, and the APP helps in navigating directions. The system consists of a TI MSP430G2553 microcontroller as an embedded device, two HC-SR04 ultrasonic sensors, and three mobile vibration motors. The proposed device is lighter and affordable, but the designed structure needs to be held through fingers, which is not comfortable for long-term use.

Sangami et al. [13] proposed a walking stick prototype that helps the VCP to move independently both indoors and outdoors. The walking stick is embedded with an SRF02 sonar sensor for obstacle detection, and RFID and a GPS module are used for indoor and outdoor location detection, respectively. A GSM module is also used for sending alert messages to preregistered contacts.

Swain et al. [14] proposed a stick to guide the VCP on their way. The components used in the system are an Arduino Uno as a controller for processing the signal obtained from different sensors, two HC-SR04 ultrasonic sensors to detect obstacles, and an IR sensor for staircase detection. GPS-GSM modules are also used to find the location of the VCP and to share that location information with a registered contact.

Ali J. Ramadhan [15] proposed a wearable smart system to improve the independent and safe mobility of visually impaired persons. It includes an ATmega328 microcontroller embedded with an Arduino Uno with various types of sensors. An HC-SR04 ultrasonic sensor is used to detect obstacles, an ADXL345 accelerometer is used for fall detection of the user, and a voice recognition sensor is used that detects the user’s voice when he or she needs assistance. As GSM and GPS modules are used, it can send an alert message to a registered mobile phone number, and also the location of the user can be tracked by their parents using that registered mobile phone. In this proposed system, the author’s idea is to keep the device always with the VCP without being forgotten, which may be beneficial in emergency situations. It has the features of lower body part obstacle detection and localization and communication in emergency situations. Since the system is designed to be worn on the wrist, therefore the user needs to maintain hand posture. Otherwise, it may always change the range or direction of obstacle detection, which may lead to a dilemma in detecting obstacles by the VCP.

Morad [16] designed a system called “GPS talking” to help a VCP in independent navigation. This device is basically a localization-based aid and does not have the feature of obstacle detection. It contains a GPS receiver, programmable interface controller (PIC) microcontroller, voice recorder, liquid crystal display (LCD), microphone, and headset. The GPS receiver receives the location coordinates and sends these data to the microcontroller for comparing these coordinates with the predefined coordinates. If the coordinate range is matched, then it plays a voice recorder message and notifies the user through the headset. The LCD is used to read the current position coordinates by the designer.

Kher Chaitrali et al. [17] proposed a navigation system that produces voice messages for obstacle detection and navigation. This system consists of an 89c51 microcontroller, Bluetooth, an analog-to-digital converter (ADC), an IR sensor, an RFID sensor and RFID tags, and android APP. The IR sensor detects obstacles and the RFID sensor that is fixed to the walking stick reads the RFID tags that are installed in public places and sends these data to the microcontroller to produce an alarm. Apart from this, the APP of the blind person gets this information through Bluetooth and produces vibrations or voice messages for obstacle avoidance and location navigation. Another APP is used by the user’s family members to get the latest location of the user. RFID is one of the very good solutions for navigation, but has some drawbacks, which are discussed in the work [9].

The above-mentioned systems of Section 2.1 have only obstacle detection capabilities and do not have any features for localization and communication, and the systems of Section 2.2 are position locator devices and are integrated with other sensors to extend facilities for VCPs. Since these systems provide various features and are the amalgamation of many electronic components, so they are termed hybrid ETAs.

Altogether, if we summarize, the above-discussed systems use various sensors with different techniques to detect obstacles such as ultrasonic sensors, IR sensors, and sonar sensors. Ultrasonic sensors and sonar sensors are quite related, as both sensors work based on the principle of reflected sound waves, whereas IR sensors work based on reflected light waves. IR sensors have a drawback in dark conditions, so in bright conditions they have better performance, but sometimes they may fluctuate in various light conditions and also get interfered with from sunlight. Thus, these are some limitations of using IR sensor-based devices. However, ultrasonic sensors are applicable for both light and dark conditions, and also are not affected by many other factors such as dust, smoke, mist, vapor, and lint. In addition, ultrasonic sensors have a longer range of detection compared to IR sensors. However, IR sensors are less expensive compared to ultrasonic sensors. Apart from this, in many proposed systems, GSM technology has been mentioned for communication. GSM is a worldwide technology, but it has certain limitations, such as bandwidth lag, so with multiple users the transmission can encounter interference. GSM is second-generation technology, but now already faster technologies such as 3G, 4G, and 5G have developed in different networks. Again, it may interfere with certain electronic devices, such as hearing aids. As we can see, some of the proposed systems are wearable, and it is good that the hand of the VCP is not occupied. However, according to our observations, a stick is more important for a VCP: If unfortunately any of the sensors fail, the stick can prevent direct collision with obstacles. A VCP walking is different from a normal person, as human eyes cannot be completely substituted by any other substitution, so a stick can be a good supporter along with the sensors. Again, using the stick, the VCP can have a basic idea about the path, such as if the VCP’s stick is in front of him/her and there is no terrain, it means a pit.

Thus, in this paper we propose a hybrid ETA that provides multiple beneficial features. In this system, we combined a microcontroller with a single board minicomputer Raspberry Pi to make it more reliable, powerful, and efficient. Raspberry Pi works as the master controller, whereas a PIC microcontroller acts as a slave controller, and simultaneously they perform and achieve multiple features. The ETA is a sensor-based walking stick, along with APPs. It has the capability of long-range obstacle detection up to 6 m ahead of the VCP for both upper and lower body parts, and also water puddle detection. Again, we did not use extra components such as an analog-to-digital converter (ADC) for this, as the used microcontroller has an inbuilt feature of ADC. In addition, it has the features of localization and communication in emergency situations. For communication, an APP is used using a faster-speed network instead of an additional component. The emergency APP has not only the features of call forwarding and SMS sending, but also a remarkable feature for partially sighted people. The APP is camera-enabled and captures the location image with the address automatically when the cell phone is shaken, and then an option pops up to share this image using social media with friends or parents. This means a fewer number of components are used in this system to make it simple, light in weight, and affordable.

## 3. Proposed System

### 3.1. System Architecture

To overcome certain limitations of existing devices, the proposed system had to attain some requirements, such as the components of the device had to choose low cost with better accuracy to make the system affordable and reliable. The device should have the capability of integrating multiple sensors. It needed to have Wi-Fi connectivity options. In addition, the device should have the capability of connecting to a database, and the system should have the capability of being accessed remotely. Thus, considering the above requirements and the comfort zone of visually challenged people (how they can carry a walking stick easily), we tried to design this walking stick. This system is an integration of both software and hardware, as it consists of a walking stick and android-based applications. In order to achieve these requirements, we brainstormed to select suitable components for this system. There were different kinds of prototyping boards available in the market with different features and usages, such as Raspberry Pi, Arduino, BeagleBone, and PCDuino [18]. As discussed in the previous section, many of the proposed systems use Arduino as the controller, but for this system, Raspberry Pi and a PIC microcontroller were selected as the control kernel. The reasons for selecting Raspberry Pi and PIC are explained in the following paragraph.

**Figure 1:**
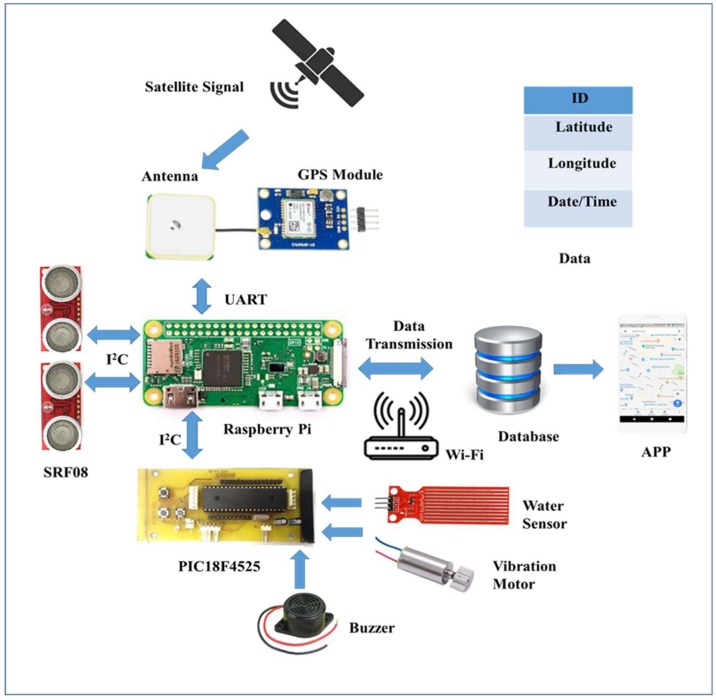
Proposed system architecture.

Raspberry Pi is a fully functioned single-board computer, a system-on-chip (SOC) device, that runs on a Linux operating system, and the operating system can be easily switched by just swapping the memory card, as it uses an SD card to install the operating system. The Arduino is not as powerful as the Raspberry Pi in terms of audio, video, or internet features. The main advantage is that Raspberry Pi can do multiple tasks at a time, like a computer. Raspberry Pi is 40 times faster than Arduino and can be accessed remotely via secure shell (SSH). For only hardware-based projects, Arduino is the best choice, but for software applications where networking, databases, and webservers are involved, then Raspberry Pi is the better option. Low power consumption and low cost makes the Raspberry Pi efficient [19,20,21]. In addition, it offers a number of advantages, such as being good at computing and allowing different options for continuous information transmission [22]. In addition, the newly developed version of Raspberry Pi has pulse width modulation (PWM), and it does not have only analog inputs natively, but the Arduino and PIC microcontrollers do. As Raspberry Pi is the main controller of this system and is capable of doing computation, networking, and transmission efficiently, we chose a PIC microcontroller instead of choosing another prototype, Arduino, to add an ADC feature to Raspberry Pi. In addition to this, it was efficient for this project due to its low cost and being smaller in size [23]. The summarized list of comparisons between Raspberry Pi, the PIC microcontroller, and Arduino are given in Table 1. The architecture of the system is shown in Figure 1. The materials used in this system are described below in detail.

#### 3.1.1. Control Kernel

Raspberry Pi acts as a master controller and PIC18F4525 as a slave controller, together making this system useful in achieving multiple applications.

##### Raspberry Pi

Raspberry Pi is basically a credit card-sized single-board computer developed in the United Kingdom by the Raspberry Pi foundation. From time to time, several generations of Raspberry Pi are developed with more advanced features, such as memory capacity and peripheral device support. In this paper, we used Raspberry Pi Zero W v1.1 (Adafruit, New York City, NY, USA). It was used due to its minimized size compared to Model A+ and its lighter weight. This is the advanced version of Raspberry Pi Zero, as it has an inbuilt BCM43143 Wi-Fi chip to provide 802.11 wireless LAN like the Raspberry Pi 3 Model B and Bluetooth 4.1/BLE. It has a 1-Ghz single-core CPU, 512 MB RAM with a Micro SD slot, a Hat-compatible 40 pin header, a mini-HDMI port, two micro-USB ports, and a CSI camera connector. It allows a number of programming languages to be used by the user, such as Python, C++, C, and Java language. It can communicate with the slave controller through I2C protocol.

##### PIC Microcontroller

Here, an advanced microcontroller PIC18F4525 (Microchip Technology Inc., Taipei, Taiwan) is connected to the Raspberry Pi through I2C as a slave controller to accelerate the response speed of Raspberry Pi. A printed circuit board (PCB) was designed, where the microcontroller is embedded and mechanically supports and electrically connects with other electrical components. Therefore, it is easy and flexible for the microcontroller to control and access the other components without cables. This microcontroller has 48 K flash memory, DC-40 MHz operating frequency, and a 10-bit analog-to-digital converter. For this, an MPLAB C18 compiler was used. The compiler is a 32-bit Windows console application that is fully compatible with MPLAB IDE (Integrated Development Environment), and it allows for debug source code using an MPLAB ICD 2 in-circuit debugger. The MPLAB ICD 2 is connected using a USB between the PC, operated with MPLAB IDE, and the target PIC18F4525 microcontroller. Therefore, the output of sensors in the form of digital data can be watched directly in the MPLAB watch windows of the MPLAB IDE software interface.

#### 3.1.2. Sensors

##### Ultrasonic Sensor

One of the major functions of this project was detecting obstacles in the way of visually challenged people. There are many ultrasonic range finders available with different features in the market. In this project, two SRF08 ultrasonic sensors (SRF08 ultrasonic sensor, Active Robots Limited, Chilcompton, UK) are used for detecting obstacles and are interfaced with the Raspberry Pi through I2C communication. The ultrasonic sensor generates frequencies above 20 KHz, which is higher than the audible range of a human being, so it is not harmful to human beings. Then, the ultrasonic sensor looks forward to receive echoes from the sound waves bouncing back off any obstacles. If an echo wave is received, then it calculates the distance to the obstacles from the ultrasonic sensor by the total time taken by the signal to get transmitted and return back. The main advantage of using ultrasonic sensors over IR sensors is that they can detect any objects that have the property of sound reflection, such as metal, nonmetal, transparent or colored, and liquid. Compared to other ultrasonic sensors (SRF05, SRF02, HC-SR04), they give a better range of detection, from 3 cm to 6 m, which was a better solution for this project so that obstacles can be detected and collisions avoided in time. In addition, the SRF08 is able to detect obstacles in front of it as well as in a conical shape of 45 degrees.

##### Water Level Sensor

The water sensor is used to detect small water accumulation or puddles on the walking path. Here, a Funduino water level sensor (Hobby Components Ltd., Hollingwood, UK) was used. It is a simple, portable, inexpensive, compact, lightweight, low power-consuming, high-sensitivity, and high-level/drop recognition sensor. The sensor has a series of parallel wires that are exposed traces to measure the volume of water or detect water droplets. The module has three pins: + (5V), − (GND), and S (signal). Thus, it can easily connect to a microprocessor, PIC microcontroller, Arduino controller, advanced virtual RISC (AVR) microcontroller, STC microcontroller, and others. Here, the sensor is connected to the analog pin of PIC18F4525. As it is an analog-type sensor, the analog output is converted into a digital signal using an ADC of PIC18F4525.

#### 3.1.3. Other Components

##### GPS Module/Global Positioning System

GPS is a satellite-based radio navigation system owned by the United States Government and operated by the United States Air Force. It provides geolocation and time information to a GPS receiver in all weather conditions, anywhere on or near the earth where there is an unobstructed line of sight to four or more GPS satellites. It is free of cost to access for those who have a GPS receiver. Here, a u-blox NEO-6M GPS module (u-blox, Taipei City, Taiwan) was used. It is interfaced with the Raspberry Pi and receives signals from the satellites. Due to being cost effective and having low power consumption, compact size, and memory options, it was suitable for this project. For serial communication, the transmission mode is RX and TX through the universal asynchronous receiver transmitter (UART) interface.

##### Vibration Motor

Here, a vibration motor is used to inform the user about an obstacle detected by the ultrasonic sensors. A cylinder-type vibration motor (Taiwan Intelligent Sensing Technology, Tainan City, Taiwan) is used. The motor is connected to the PIC18F4525 microcontroller so that it can be programmed to control the motor speed. The output of the vibration motor is controlled using a PWM to obtain different vibration patterns, so that the VCP can identify the type of detected obstacles.

##### Buzzer

An electronic buzzer (Ariose Electronics Co., Ltd., Taoyuan City, Taiwan) is used to inform the user about detecting water accumulation on the path of the user. It produces a beep sound when the water level sensor touches the water surface. In this system, the buzzer is connected to the PIC18F4525 microcontroller.

##### Power Supply

A portable dual USB 5V/2A/1A, 10400 mAh universal battery power bank (Yoobao, Shenzhen, China) is used for the power supply. The charge can last at least 6 h on continuous use, and the battery charge can last for 46–47 h on standby.

### 3.2. System Implementation and Execution

**Figure 2:**
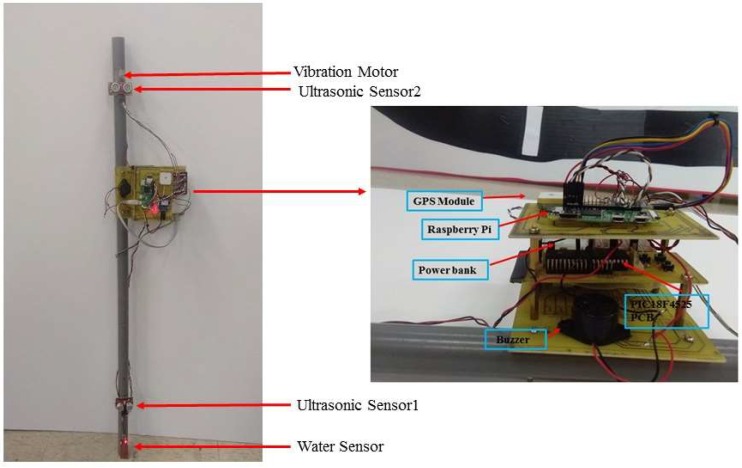
Real design of proposed walking stick.

This is a Raspberry Pi and PIC-based intelligent walking stick that is integrated with obstacle detective sensors, a location information receiving module, and Android-based applications that are used to get the location information of the user as well as being helpful in emergency situations. We designed the walking stick, as shown in Figure 2. A polyvinyl chloride (PVC) pipe was used for making this stick, as this material is easily available and also easy to design. Ultrasonic sensors, a water sensor, a GPS module, Raspberry Pi, a PIC18F4525 microcontroller, a vibration motor, and a buzzer were all fixed to the walking stick. A three-layer structure was designed, where controllers, the GPS module, the buzzer, and the power bank are placed, which is shown in Figure 2. The water sensor is placed at the bottom of the stick. One ultrasonic sensor is placed at a height of 17 cm and another is at a height of 92 cm from the bottom. The vibration motor is placed near the handgrip of the stick.

**Figure 3:**
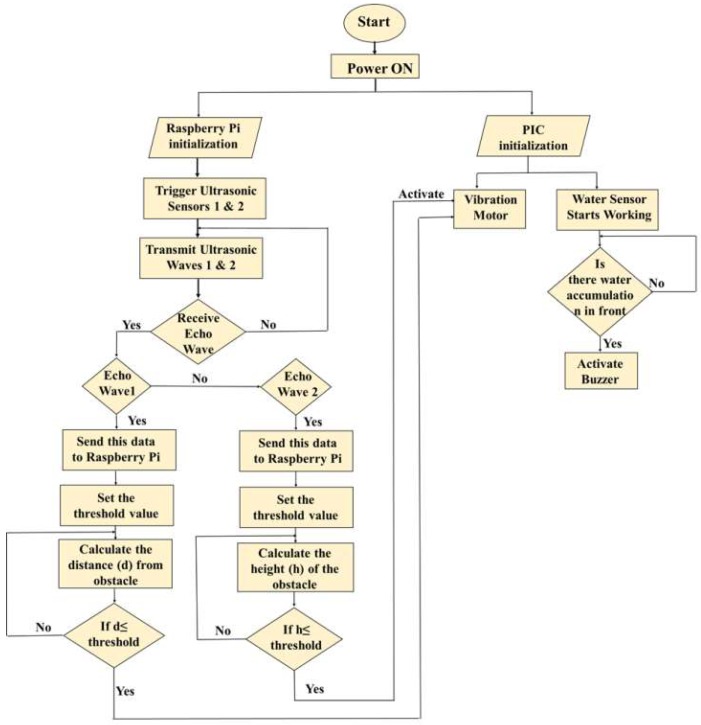
Flowchart of execution of obstacle detection.

The system execution process is divided into two parts. One is the obstacle detection part, and the other is for receiving location information. When the power supply is given to Raspberry Pi, the whole system is initialized and starts working. The flow chart of the execution of the obstacle detection function is shown in Figure 3 and is explained in the following paragraphs.

The ultrasonic sensor follows the same principle as a bat uses to follow to find its prey, called the echolocation principle. The ultrasonic sensor emits an ultrasound wave at the normal speed of a sound wave beyond the human audible range and waits for the echo wave to be received. Usually, the speed of the sound wave in air is 343 m/s at normal room temperature. The wave travels away from the sensor in a conical shape, and the sound bounces back to the sensor from any object in the path of this wave. After transmitting the wave, the ultrasonic sensor pauses for a while and waits for the echo wave to receive. In this system, both the ultrasonic sensors are connected to the Raspberry Pi. If the echo wave is received, then it indicates the obstacle is detected. Then the sensor sends the data to Raspberry Pi so that it can compute the distance to the object based on the elapsed time between the transmitted signal and the received signal. According to user convenience, the threshold values are set in the Raspberry Pi coding to make the visually challenged people aware about the obstacles. The threshold value is a predefined distance range within which the obstacle can be detected, and this threshold value can be changed according to user convenience. For ground level and upper body part obstacle detection, the threshold values are 200 cm and 100 cm, respectively. Then, according to the written code in Raspberry Pi, the calculated distances (*d* and *h*) are compared to the threshold value, and if the calculated distances are less than or equal to the threshold value, then the master controller Raspberry Pi gives a command to the slave controller PIC18F4525 to activate the vibration motor.

Then, the code in the PIC18F4525 is executed and makes the vibration motor ON. If the ground obstacles get detected, the motor vibrates continuously at high speed, but for upper body part obstacles, it slows down and vibrates two times in a second. A water level sensor is also used in detecting small water puddles. When the module comes in contact with water, the connected buzzer to the microcontroller produces a sound to alert the user.

**Figure 4:**
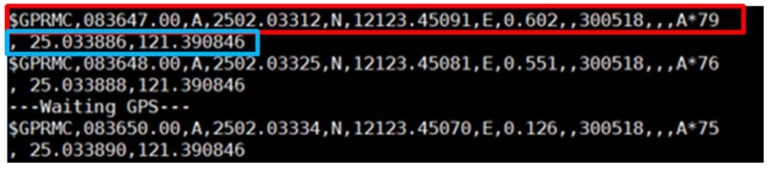
GPS output showing in NMEA format along with the converted coordinates.

Other than obstacle detection components, a GPS module is used that provides location information and helps in real-time tracking. The GPS module is connected to Raspberry Pi through a UART interface, and while the user is outside his/her home, the GPS receiver receives the signal from satellites. The output of the GPS receiver has many sentences, such as GPGGA, GPGSA, GPGSV, GPGLL, GPRMC, and GPVTG. But for this project, we needed only GPRMC, so a code was set to show only a GPRMC sentence, which is shown in Figure 4. These sentences are in National Marine Electronics Association (NMEA) format, and they contain location information in an American Standard Code for Information Interchange (ASCII) message. The latitude and longitude obtained from the $GPRMC are in degree minute format, which cannot be used directly to get location information. For this, it needs to convert into decimal degree format. Thus, a code was written in Raspberry Pi that converts these coordinates into decimal degree format automatically and sends them to the database to store and for future purposes. The explanation of one of the GPRMC sentences [24,25] from Figure 4, along with the conversion of coordinates, is discussed below.

Here, $GPRMC is a recommended minimum specific GPS that specifies the type of sentence: 083647.00 is the coordinated universal time (UTC), which would be 08:36 and 47.0 s in UTC. To convert from UTC to local time simply requires knowing the time zone of the user’s locality. The next letter represents the signal status (A-active, V-void). “A” indicates that the GPS module is receiving the signal and is working properly. “V” indicates that the GPS module is not getting the signal. Here, 2502.03312, N is the latitude at 25° 02.03312’ in the northern hemisphere, and 12123.45091, E is the longitude in 121° 23.45091’ in the eastern hemisphere. The latitude and longitude are in degree minute format and cannot be used directly on Google Maps. Thus, these values need to be converted into decimal degree format by simply realizing that one degree equals sixty minutes. Thus, for this case, if we want to do it manually, then 02.03312 min = 02.03312/60 degrees = 0.033885333 degrees, and thus latitude is 25.03388533, and for 23.45091 min = 23.45091/60 degrees = 0.3908485 degrees, longitude is 121.3908485. Here, 0.602 is the speed in knots; 300518 is the date in DD/MM/YY, so here it is 30 May 2018; and *79 is the checksum data. The last two numbers of the above sentence represent the converted latitude and longitude in decimal degrees, respectively. Here, this conversion is done automatically according to the written programming.

**Figure 5:**
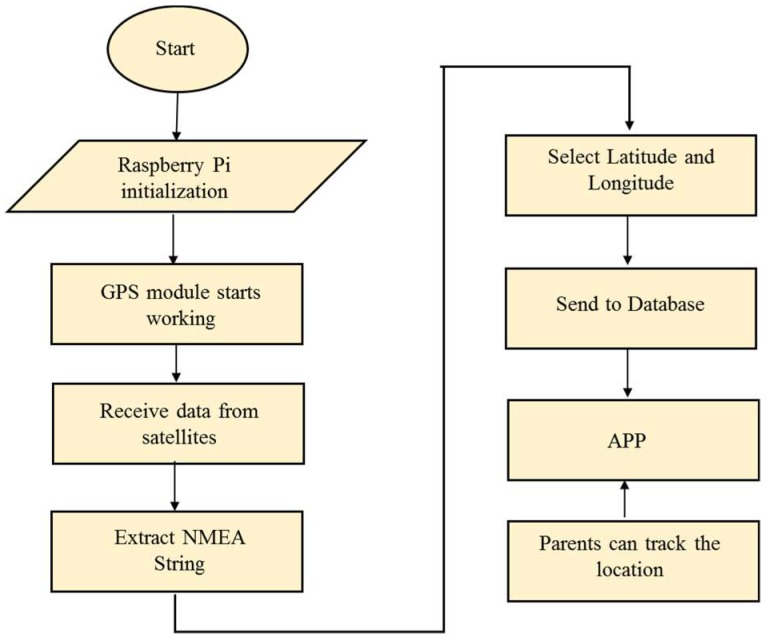
Flow chart of getting location information.

For database implementation, we used XAMPP software, which is a free and open source cross-platform web server solution by Apache Friends. Here, the SQL, PHP, and JavaScript programming languages were used. Later, these coordinates were used to get the location information of the user using an Android-based application. The flow chart of the workings of the GPS module providing location information is shown in Figure 5.

## 4. Results

### 4.1. Obstacle Detection

**Figure 6:**
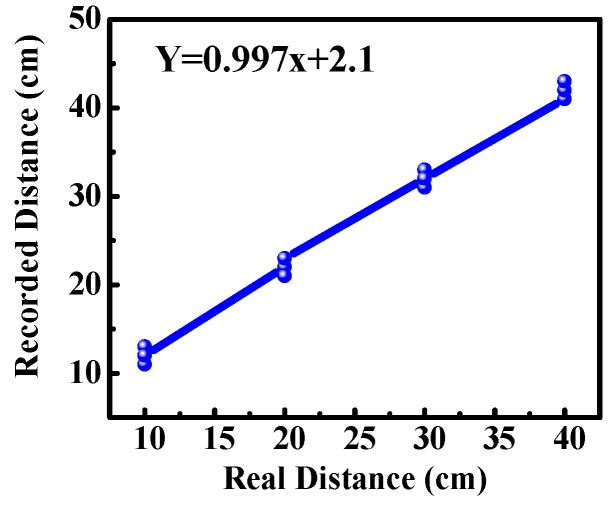
Comparison of distance between real value and recorded value.

**Figure 7:**
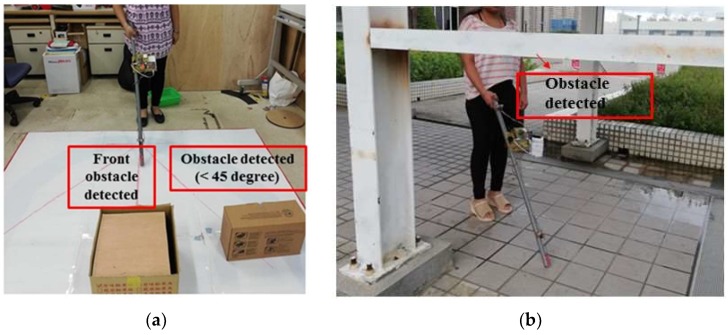
Experimental result of obstacle detection: (**a**) Indoor testing; (**b**) outdoor testing.

For obstacle detection, the system was experimented on in our lab and inside our campus. The result was found that ultrasonic sensors were able to detect obstacles accurately, and the measured distance to the obstacles was also approximately correct. For comparing the obtained distance to real distance, a graph was plotted between the recorded value and the real value, as shown in Figure 6. The graph shows a linear curve that indicates the error in distance measurement was small. According to the SRF08 ultrasonic range finder datasheet, it could detect obstacles in a conical shape of a range of 45 degrees. We experimented with the beam pattern of SRF08, and the result was found that the ultrasonic sensor could detect the front obstacle as well as in between the range of a conical shape of 45 degrees and up to a height of 30 to 40 cm from ground level, as shown in Figure 7a. Similarly, the second ultrasonic sensor could detect the obstacles at a height of 130 to 140 cm. For example, in certain places the pedestrian had a side wall that seemed to be no obstacle from the ground, as it was vacant from below, but at a certain height had an iron bar, so the second ultrasonic sensor was helpful in detecting this kind of obstacle, as shown in Figure 7b.

### 4.2. Location Information

**Figure 8:**
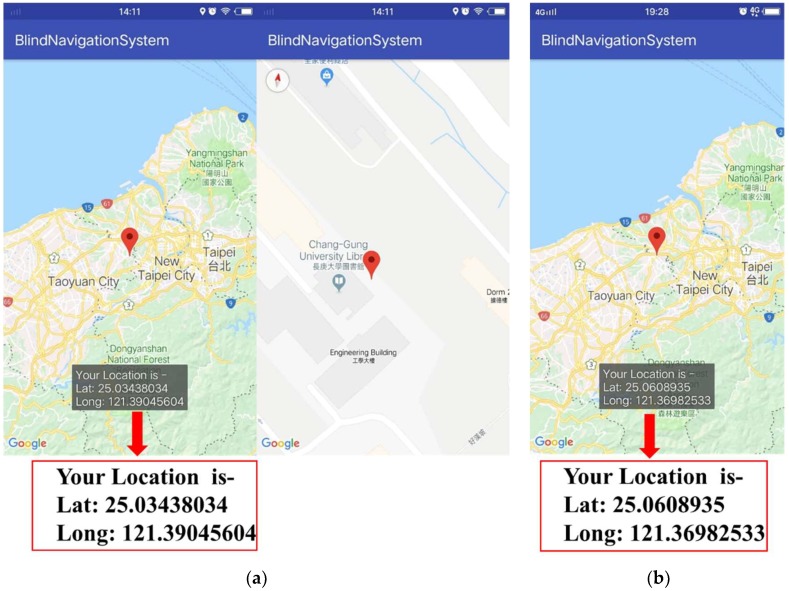
APP showing the user location at (**a**) Chang Gung University library; (**b**) Taoyuan Airport MRT A8.

The application was designed using Android studio and Google Map API, which provides user location, so that the parents of the user can locate the user. For this, the experiment was done while the user was roaming around inside and outside the campus. The APP showed the latitude and longitude of the location written on the map. Figure 8a,b shows the location of the user near the library of Chang Gung University and near Taoyuan Airport MRT A8 (Chang Gung Memorial Hospital station), respectively.

### 4.3. Panic Situations

**Figure 9:**
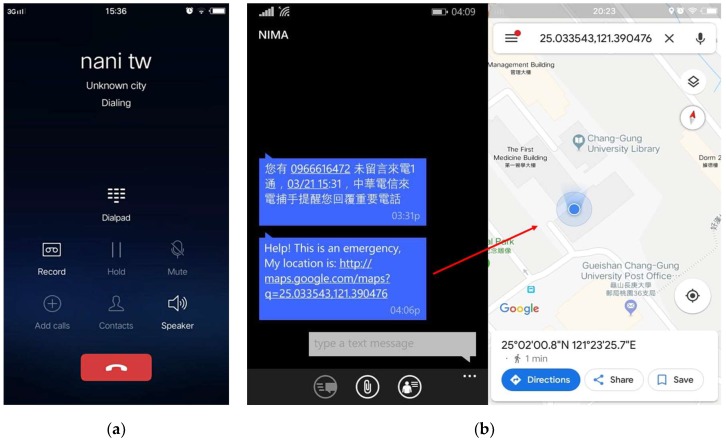
APP showing (**a**) emergency call forwarding to a primary contact and (**b**) a sent emergency SMS to an emergency contact.

**Figure 10:**
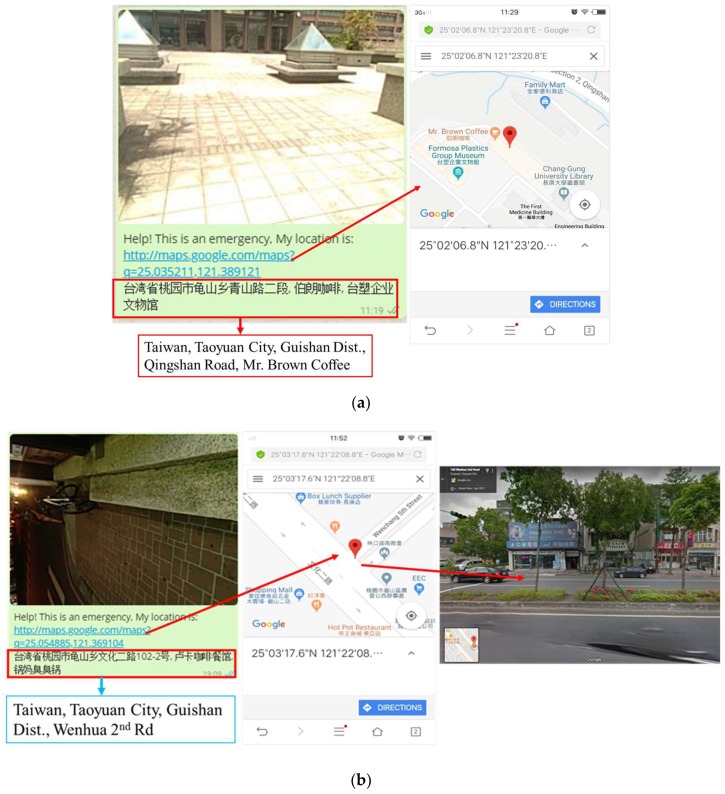
APP showing a shared picture with location addresses: (**a**) Mr. Brown Coffee, Chang Gung University; (**b**) in front of a cell phone shop at Wenhua 2nd road, Taoyuan.

In some situations, visually challenged people face many difficulties in an unfamiliar environment or have some health issues and wish to contact their parents or friends immediately. Usually VCPs have to depend upon others for help, but the emergency application is very useful in these situations. In this system, an Android-based emergency application is used that is downloaded from the open source Google play store [26]. After testing it in several situations, it was found that this APP is really helpful for both blind as well as for partially sighted people. Features of this APP include:

- An emergency SMS and call can be forwarded to emergency contacts that are saved to the APP by shaking the user’s cell phone or pressing the power button four times in 5 s. An SMS can be sent to all emergency contacts, but the call can be forwarded to only one contact, which is saved as the primary emergency contact. The sensitivity of shaking detection can be adjusted according to the user’s requirements. The SMS contains the latitude and longitude of the location, along with the link to Google Maps. Thus, by clicking on this link, it redirects to Google Maps showing the precise location of the user. A demo of call and SMS forwarding in an emergency to primary and other emergency contacts is shown in Figure 9a,b;
- It also captures the location image with a detailed address when shaking the cell phone automatically. After that, it shows options on social media to share a picture with friends or relatives so that the user’s friends or relatives can get a better idea by looking at the image in order to find the exact location of the user in an emergency. This feature can be applicable to partially sighted people. The image sent to friends through social media using this emergency application is shown in Figure 10. The address in Figure 10a,b represents locations near Mr. Brown Coffee, Chang Gung University, and Wenhua 2nd road, Taoyuan city, in front of a cell phone shop, respectively;
- On the first page of the APP, it always displays the real-time location of the user, which is helpful for partially sighted people, and also there is a siren button at the center of the page. In certain emergency conditions, if the user touches this button, a sound is produced informing the surrounding people that the user needs help.

## 5. Discussion

First of all, each component was experimented on individually by running the code for each function to analyze the results and performance. Then we combined the codes of each function, executed them simultaneously, and integrated all the components into the walking stick for implementation. Like the other systems that use an emergency button or GSM or other components for communication with family members, this system also includes this feature. However, we did not use any additional modules for this, but rather used a useful emergency APP. This was found to be very convenient, as the call can be automatically forwarded by just shaking the cell phone, so the user can easily communicate with a family member, and an SMS can also be sent. Mostly to reduce cost and complexity, we tried to use a few electronic components smaller in size. In the future, we want to collect data regarding the types of obstacles detected by the user, and data analysis will be done using a neural network learning algorithm to predict any dangerous situations encountered by the blind person, so that they can be avoided in the future.

## 6. Conclusions

This system was designed comprising a walking stick and an APP, where the control kernel is performed by Raspberry Pi. The embedded system is implemented in the stick and succeeded in integrating the ultrasonic sensor, water level sensor, vibration motor, buzzer, GPS module, Raspberry Pi as the master controller, and the PIC18F4525 microcontroller as a slave controller in the system. All the components worked well and provided accurate data from the sensors. As the walking stick is embedded with two ultrasonic sensors, it can detect obstacles at the ground level, as well as for upper body parts. The created APP is useful for parents tracking user location, and the emergency APP is very useful for the user in panic situations. Thus, ultimately this could increase the confidence level of the user as well as make him/her feel secure.

## Figures and Tables

**Table 1:** Comparison between Raspberry-Pi, the PIC microcontroller, and Arduino [18,19,20,23].

Points of Comparison	Raspberry Pi	PIC Microcontroller	Arduino
Base Price in US $	35 (For this system 10$)	2~10	20
Operating Voltage	5V	5V	5V
Power Consumption	~150 mA	~20 mA	~50 mA
Operating System	Linux	Custom	Custom
Memory	512 MB RAM, Micro SD Card Slot	48 KB Flash Memory	32 KB Flash Memory/2KB SRAM/1KB EEPROM
I/O Pins	40 GPIO Pins (8 Digital, 0 Analog)	28~44 Pins (For this system 40 Pins, 13 ADC Input channels, 10 bit)	14 Digital (6 PWM Output), 6 Analog
Peripherals	Dual USB connector, 1 Micro USB Power, Video and Audio Output, 1 Ethernet	None	None
Internet	Yes	Via shield	Via shield
Dimension	8.6 cm × 5.4 cm × 1.7 cm (For this system 65 mm × 30 mm × 0.2 mm)	Varies according to type (For this system 5 cm × 1.3 cm × 0.7 cm)	Varies according to version (Atmega 328P Arduino, 68.6 mm × 53.3 mm)
Better Operation for	Software	Hardware	Hardware
Communication Mode	I^2^C, UART, SPI, 802.11n Wireless LAN, Bluetooth 4.1/BLE	I^2^C, UART, SPI, PSP	UART, SPI, I^2^C
